# ‘Is this knowledge mine and nobody else's? I don't feel that.’ Patient views about consent, confidentiality and information-sharing in genetic medicine

**DOI:** 10.1136/medethics-2015-102781

**Published:** 2016-01-07

**Authors:** Sandi Dheensa, Angela Fenwick, Anneke Lucassen

**Affiliations:** 1 Clinical Ethics and Law, Faculty of Medicine, University of Southampton, Southampton, UK; 2 Wessex Clinical Genetics Service, University Hospital Southampton NHS Foundation Trust

**Keywords:** Autonomy, Genetic Screening/Testing, Confidentiality/Privacy, Family, Genethics

## Abstract

In genetic medicine, a patient's diagnosis can mean their family members are also at risk, raising a question about how consent and confidentiality should function in clinical genetics. This question is particularly pressing when it is unclear whether a patient has shared information. Conventionally, healthcare professionals view confidentiality at an individual level and ‘disclosure without consent’ as the exception, not the rule. The relational joint account model, by contrast, conceptualises genetic information as confidential at the familial level and encourages professionals to take disclosure as the default position. In this study, we interviewed 33 patients about consent and confidentiality and analysed data thematically. Our first theme showed that although participants thought of certain aspects of genetic conditions—for example, the way they affect day-to-day health—as somewhat personal, they perceived genetic information—for example, the mutation in isolation—as familial. Most thought these elements were separable and thought family members had a right to know the latter, identifying a broad range of harms that would justify disclosure. Our second theme illustrated that participants nonetheless had some concerns about what, if any, implications there would be of professionals treating such information as familial and they emphasised the importance of being informed about the way their information would be shared. Based on these results, we recommend that professionals take disclosure as the default position, but make clear that they will treat genetic information as familial during initial consultations and address any concerns therein.

## Introduction

Healthcare professionals (HCPs) have a duty to safeguard individual patients’ rights and interests: keeping their medical information confidential protects privacy and engenders trust, while asking consent to share that information protects their autonomy. Individual rights and interests, however, are often intertwined with those of others, which is especially so in genetic medicine, where one patient's diagnosis can indicate that relatives are also at risk.^1^ This raises a question about how consent and confidentiality should function in genetic medicine. For example, should HCPs share information with patients’ at-risk relatives if they seem unlikely to do so themselves? In such situations, HCPs need to balance their patient's privacy and (ostensibly) autonomous decision not to share with the potential to prevent harm to the relative. One study suggests explicit refusal to share is rare in the UK,^2^ but cases discussed at the UK Genethics Forum^3^ suggest that HCPs regularly encounter and are uncertain how to manage cases where they are unsure that the patient has shared.

UK guidelines^4^ state HCPs can disclose personal information if a patient has explicitly refused consent when ‘the benefits to an individual or to society of the disclosure outweigh the public and the patient's interest in keeping the information confidential’. Other UK guidelines^5^ state that HCPs should consider the severity and predictability of the condition, while US guidelines^6^ suggest that the risk should be serious, imminent, foreseeable and likely. The default position, if relying on such guidelines and on existing norms, is for HCPs to treat patient information as confidential to them and disclosure without consent as the exception, not the rule.

Arguably, this approach to confidentiality and autonomy is based on an inaccurate conceptualisation of patients as separate from others, free from social or familial constraints. By contrast, relational approaches to autonomy stress that patients develop their autonomy through social embeddedness and engagement with others; that one person's choices affect other people's autonomy; and that despite wanting to protect their personal interests, people are interested in maintaining family and community relationships. Relational approaches are rooted in communitarian and feminist ethics and underscore relatives’ moral responsibilities to each other.^7^

Along these same lines is the joint account model of confidentiality, where genetic information is conceptualised as familial and ‘belonging’ to all the relatives it might affect, not just the individual in whom it was first identified. One reason the information is seen and treated as familial is that HCPs likely initiated that patient's test because of familial information provided without relatives’ explicit consent. The default position here is for HCPs to share information that is relevant to at-risk relatives unless they identify circumstances that would justify excluding other ‘account holders’ from their information.^8^ The joint account model informs current guidelines from the British Society for Genetic Medicine (BSGM) about consent and confidentiality. The guidelines recommend HCPs use a consent form for genetic testing that includes a statement such as, “I acknowledge that my results will sometimes be used to inform the appropriate healthcare of family members” instead of asking patients to agree or disagree to sharing.^9^ Others too have suggested HCPs make clear at the outset they will treat information as familial.^10^ ^11^

Foster *et al*^12^ have recently suggested that a relational approach together with the joint account model (relational joint account herein) would be a workable model in the courts. Notwithstanding, some have pointed out unresolved issues, including the fuzzy boundaries of the ‘genetic family,’ and their competing rights to know and not know^13^ ^14^ It is unclear what patients think, because existing empirical studies about consent and confidentiality are few and limited: they have explored the issues only briefly; used surveys that lack the nuances of qualitative research; focused narrowly on situations where patients explicitly refuse to share; and have questionable transferability to the UK and National Health Service (NHS).^15–19^ Patients’ views are timely and important to consider, because genetics is rapidly moving in to the genomic era, meaning the likelihood of needing to involve family members to clarify a patient's uncertain result or because of an incidental/additional finding with relevance to relatives will increase.^20^ Our overall aim therefore was to explore patients’ views, in particular about the relational joint account, and identify implications to policy and practice.

## Method

We sent information about our research with a reply slip to collaborators in three large genetics centres in the UK, who posted the information onwards to all recent patients seen for hereditary cancers and cardiac conditions in the previous 2 years. Online supplementary figure S1 shows how the project was framed on information sheets: we took care to keep the information general so as not to ‘lead’ participants into one view or another or bias recruitment towards people who supported information sharing. Interested parties could return a reply slip to SD in a stamped addressed envelope. We also posted information on online condition-specific support groups. SD contacted those who sent back a slip or responded online to arrange a suitable time, date, and, for face-to-face interviews, location. We asked participants to choose somewhere they could speak with us undisturbed to keep discussions as confidential as possible.

Discussions about the research topic when we were arranging the interview were kept general: we refrained from detailing any arguments about our topic so that participants would later share their views and experiences with minimal influence from the researchers.

This was important because, as an ‘instrument’ of the research, the qualitative researcher can introduce bias in data collection and analysis, thus affecting the trustworthiness of the findings. We took several steps to minimise this possibility.^21^ First, our interview schedule comprised of general, open-ended, and non-leading questions designed around our research questions and empirical and conceptual literature. Second, we piloted the schedule in the first interview, after which we discarded unnecessary, complex and potentially directive questions. SD conducted all the interviews to retain consistency in the questions asked across participants. Thirdly, AL and AF analysed sections of data independently to enhance reliability and rigour, and these analyses were discussed and compared. Another way we limited bias was to frame the purpose of the project and summarise its aims in a non-leading way in the preamble to each interview (see online supplementary figure S1). Since data collection and analysis were iterative, we were able to identify new and important questions and areas of ambiguity or tentativeness in the analysis that could be explored in subsequent interviews. We used general scenarios about non-disclosures to prompt discussion.

In total, we conducted semistructured interviews with 33 adult participants from England. Interviews lasted around 1 h and were face-to-face (n=25), by phone (n=5) or Skype (n=3). Data were analysed thematically.^22^ Although one participant had been tested for Huntington's disease, we focused in interviews on views about conditions conventionally thought of as amenable to medical intervention.

## Results

Table 1 contains details about participants. We identified two major themes: the first about the nature of genetic information and the second about controlling its flow. Online supplementary table S2 contains additional supportive quotations^i^.

**Table 1:** Participant details

Sex	22 female (F); 11 male (M)
Condition	Hereditary breast/ovarian cancer (HBOC) (n=14: n=5 had cancer before test) Lynch syndrome (n=8: n=6 had cancer before test) Familial adenomatous polyposis (FAP) (n=3: n=1 had cancer before test) Alport syndrome (n=4) Hereditary cardiomyopathy (n=2) Hereditary haemochromatosis (n=1) Huntington's disease (n=1)
Test result	All tested and diagnosed as positive except n=2 tested negative (P9,cardiomyopathy,F; P11,Huntington,F) n=2 untested (P5,Lynch,F; P12,HBOC,F)
Learning about risk	Most learnt of their risk at the same time as siblings and other close relatives. Seven were the first to be tested and told family about their risk immediately, often before having the test/getting their result
Disclosure	None had withheld information although a few had not told distant relatives yet, mostly because they had no contact details. Three participants’ relatives did not share information about risk with them. P5 (possible Lynch,F): cousin was withholding his exact mutation so she could not have a definitive test. P18 (HBOC,F): sister did not want to tell her about risk directly so asked her General Practitioner (GP) to do so. P18 found out months later as GP failed to pass the message on. P30 (HBOC,F): sister did not share her HBOC diagnosis. P30 found out because a nurse mentioned it during an appointment with the affected withholding sister, which another sister attended. The latter shared the information

### Genetic information should be disclosed

#### Genetic information is not personal

Almost all participants saw genetic information as essentially familial, with several saying something akin to “this isn't my information, I don't own the gene” (P16, hereditary breast/ovarian cancer (HBOC),F). Participants perceived relatives as having a right to know about their potential risk and themselves as having a ‘duty’ (P12,possible HBOC,F) to help them by sharing;

P13:[The information is] only private maybe to the family, but not particularly private to me. I think it's probably my family's right to know about it, so they should be informed about it, and it's not my decision to keep that from them. Outside of the family then probably yes[it is private], but within the family or the wider family, then no. (Cardiomyopathy,M)

The view that genetic information is private at a familial rather than individual level and the perceived duty to consider the interests of the family aligns with the relational joint account. Participants said their views would be unchanged even if they were estranged from their at-risk relatives. Several said a patient's refusal to share information would be selfish, with another saying it would be irresponsible and another ‘a betrayal’. They also felt genetic information should be used to progress research and help humanity, not just family.

#### A tricky situation, but confidentiality is ‘by-the-by’

Some participants talked about how although genetic information was familial, it was simultaneously ‘personal’. For example, P7 (Alport,F) distinguished between her condition and the gene that caused it: Alport syndrome's effect on her was personal, but the genetic information in isolation was not—nor was it owned by her. This perception hints that the two types of information (personal and familial) were linked, but separable. However, some participants—particularly those who had experienced non-disclosures in their family—saw this personal-familial duality as a problematic tension. For example, P30 (HBOC,F), whose sister had kept her diagnosis a secret was unsure whether and how HCPs could practically and ethically share the relevant information;

P30: I felt as if that blood should have been everybody's, but I know it's her blood and it's her result, but I felt like that result should then have gone into a bank for any people that may be affected by it. But I don't know whether that would ever be something that you could, because obviously it's not my blood, it's her blood. I think there needs to be a way—because there's too many people out there in my situation—where if they are doing the test that it becomes public for the family. (HBOC,F)

The separation of personal and familial information was difficult to imagine because her sister was the tested person and had refused to share information. P30's uncertainty appeared to stem from sensing that her sister might have ‘owned’ the genetic information, or had some other special right over it. The reason for this intuition was that the result was generated by doing a test on her sister's body, with her consent and cooperation, and was contained within her blood. Brock^23^ has highlighted how factors such as these explain why one might think a person's result is confidential to them. But like Brock, P30 seemed to conclude that these reasons should not prevent HCPs from sharing, given the risks to relatives.

Setting aside the practical difficulties of sharing information (eg, HCPs having no contact details for relatives), participants found it generally acceptable for HCPs to share information without the tested person's explicit consent. Participants thought that this would indeed constitute a breach of patient confidence, but that this harm was trivial compared with the benefit of knowing about risk. Here, participants’ views did not align conceptually with the joint account model, under which sharing genetic information with family does not constitute a breach. Yet their views did align with the model in practical terms (ie, erring on the side of disclosure rather than confidence). Preventing illness that could lead to an avoidable death was the main justification;

P17: I think that (confidentiality) should be by-the-by when you could be saving somebody's life. They could have an illness or a potential illness that's going to kill them. (Lynch,M)

P3 (Alport,F) saw another benefit: by sharing information between relatives who were not in contact, HCPs could bring affected relatives together, who could then support each other in managing the condition. Some suggested deidentifying information before sharing, even though, as they recognised, the patient's identity could remain obvious.

P29:[At-risk relatives] should still be able to be told that it's in the family and just not who and where it's come from. [HBOC,F]

Notably, a small minority of participants laid more emphasis on the importance of HCPs encouraging patients to share information themselves. Their main reasons were that receiving unexpected information from HCPs could distress relatives (P12, possible HBOC,F; P26,HBOC,F) and disclosure could erode trust and make people reluctant to reveal information (P26). P7 (Alport,F) to an extent agreed but felt more conflicted: she thought sharing was important to protect lives, but breaching could violate the patient's ‘free will’. She described how even though her Alport syndrome had a personal effect on her life, the genetic information itself (ie, deidentified) was not personal or owned by her. Along with a few others, she highlighted the importance of HCPs supporting, encouraging and exploring what they perceived to be the likely reasons for non-disclosures: relationship or emotional problems. Crucially, none thought that HCPs should respect patients’ refusals on the basis that the information was private and personal to them.

#### Broad harms justify disclosure

A frequently cited reason for disclosure (even without consent) was that it would enable relatives to make timely choices: they thought relatives should be tested and offered treatment/intervention as soon as their risk was apparent. For this reason, most participants also thought HCPs should spend only limited time encouraging reluctant patients to tell relatives themselves. They saw risk as ‘a ticking time bomb’ (P9,FAP,F) and held this view regardless of the timing of onset. Relatedly, participants thought HCPs should share information to allow relatives to make reproductive decisions. To an extent, this view depended on the condition: P26 (HBOC,F) said conditions more ‘debilitating’ than hereditary cancer would warrant disclosure based on reproductive risk, but P24 (HBOC,F) and P9 (FAP,F) said cancer warranted it too. Either way, harm did not have to be imminent to justify disclosure without consent.

Participants moreover identified non-medical harms and benefits that they thought it important HCPs consider, such as the damage that delayed diagnosis could have on family relationships and psychological well-being. For these reasons, participants thought there could be good arguments to disclose conditions not amenable to medical intervention, such as early onset Alzheimer disease, although they perceived such situations to be more complicated because of the distress disclosure could cause. Overall, participants’ views thus aligned with the criticism of the conventional approach to breaching confidentiality, that it entails a too narrow definition of justifiable harm.^8^

### Having some control over information is desirable

#### Choice has upsides and downsides

Despite discussing genetic information as familial, participants’ attitudes towards their information were not completely permissive. Some worried that if HCPs saw and treated genetic information as familial, they would share it without the patient's knowledge, permission or consent. These participants understandably expressed a preference that HCPs tell them how their information would be used (although they generally trusted HCPs to share information appropriately).

Looking more closely at types of permission and consent, participants struggled with whether HCPs should ask patients whether they agreed or disagreed to them sharing information with relatives or whether HCPs should share patients’ information as a condition of them receiving care. Some defended using the latter, saying that asking patients’ permission would undesirably give them an opportunity to refuse. Nevertheless, this solution was pragmatic rather than optimal and there were some perceived disadvantages;

P24: In some ways it would make you feel a bit powerless, that this isn't your information to control and I feel like that wouldn't be as good. I would personally feel like ‘well this is my test, this is my information, and I should be able to tell people when and how I please’. (HBOC,F)

The perceived lack of choice made such participants defensively sway towards a view that ‘genetic information is personal’. Nonetheless, P24 continued;

P24: But I feel like at the same time it is important for other people to know and if you do get someone who (says), ‘no, it doesn't affect them’ or ‘I don't feel comfortable talking about it’, then in that case other people really do need to know, so then it would be good catch-all for them. It's very, it's difficult.

Even participants who had been ‘victims’ of non-disclosure found permissions problematic. P5 (possible Lynch,F) had said initially that HCPs should have overridden her cousin's refusal to share his exact Lynch syndrome mutation. Yet later, she said;

P5: It's your information to do with what you want. I suppose you should always have the choice[about sharing], but I wouldn't understand why you wouldn't share it.

I: But do you feel it's your information?

P5: It is, isn't it? I suppose initially it is. But then, it's not is it? Because you're part of a family who could be affected with it, so it's not your information, they should be told. It's a very difficult one. Yeah, I suppose initially it is your information.

I: What makes it yours?

P5: I suppose they've taken your blood from you, so it's yours. That's how I would think of it.

Like others (eg, P30), this participant sensed the tested person should have some special rights over the information because it came from their blood, but it was difficult to articulate the reasoning behind this intuition and identify the rights, considering the relevance of this information to relatives.

Notably, other participants who thought patients should be given an ‘agree/disagree’ option additionally went on to say any ‘disagrees’ should be overruled by HCPs, in that information should be shared anyway, making the purported choice seem like a token gesture. They thought it important that HCPs ask permission to share, but also that patients not see this as an opportunity to refuse. Participants seemed to think that by asking permission, HCPs were respecting the information's personal nature and by agreeing to share it, patients were respecting its familial nature.

Something that appeared to exacerbate many participants’ struggle with permission was a reluctance to speak for everyone. They did not feel stigmatised by their diagnosis themselves so did not think stigma was a good reason for a patient's non-disclosure, yet they realised other people might feel differently. Relatedly, P10 (HBOC,F) worried that if HCPs could share genetic information without consent, they could share other (more stigmatising) medical information without consent as well. One thing participants agreed upon was that they did not want or expect HCPs to ask permission each time before they shared information with a newly identified at-risk relative. They thought this process would delay relatives receiving care and did not want to be responsible for such delays.

#### Confusion about the law

Participants were unsure how HCPs could share information legally, for example, in line with the Data Protection Act (P23,Lynch,M). This finding raises the point that a person who withholds information might do so on the assumption that laws would prevent HCPs from sharing. One participant, who was an allied HCP in the NHS, said HCPs too worried about legality. She felt, however, that a shift towards HCPs getting permission to share early in consultations and then using information appropriately to help others was much needed;

P2: Even if it's open house, I think there will always be an individual one-to-one and that is where you need to get the consent. It's incredibly difficult for geneticists; they've been brought up with consent, consent, consent, you must keep everything quiet.[They] know something about people that is so important,[but] their own ways are going to cause them to be reluctant. You can understand that. (Alport,F)

## Discussion

We have shed light on the views of people tested for, or at risk of, hereditary conditions regarding consent, confidentiality and information-sharing in genetic medicine. Much like HCPs do in practice, participants identified and balanced the harms and benefits of HCPs sharing information. Generally, participants had altruistic tendencies and thought of genetic information as familial, which aligns with the central thesis of the relational joint account model. Overall, participants supported sharing, but noted two reasons HCPs might find it difficult to share if a patient had refused consent. First, the result had been generated from one person's blood, which might confer special rights for them over it and second, personal information (the condition and its effect on the individual) and familial information (the familial mutation) were entangled. Nevertheless, they could not articulate what special rights a tested person might have over the result that would justify non-disclosure and thought HCPs should override any refusals, disentangling and sharing information where possible. Participants also thought broad harms justified sharing, that is, not just imminent and likely, or just physical, risks. Indeed, as others have argued, criteria such as imminence and seriousness are difficult to define and too restrictive because genetic medicine deals with risk rather than certainty.^24^ In this way participants’ views were different to General Medical Council^4^ and British Medical Association^5^ guidelines and aligned more with those from the BSGM.

Our finding in support of sharing contrasts two studies in which the majority of participants disagreed that HCPs should be able to disclose patients’ test results to immediate relatives without written consent.^15^ ^16^ The reason for the difference is likely to be that both of these were quantitative studies, where participants’ views were ascertained using a single question on a survey. Our results supported three other studies where researchers sought relatively more nuanced views, albeit briefly and without reference to the relational joint account. In those studies, participants thought HCPs had an obligation to share information to protect relatives from harm and save lives.^17–19^ The right not to know was not raised in our study, even though HCPs in another study used this as a reason not to share.^25^

Based on participants’ support for sharing, we recommend a shift in current practice. That is, for HCPs at the outset, before even seeking consent, to take as their default position that they will share information (where it is clinically relevant to do so) rather than keep it confidential to one person. If the patient later refuses to disclose some information, HCPs should of course weigh this in the balance when considering whether to move away from the default position. Even then, however, we consider that HCPs might be able to disclose by separating information relevant to the family from that which is relevant only to the individual. We additionally recommend that HCPs discuss with patients at the outset that they will treat elements of the information as familial and address any apprehensions patients may have then. Such discussion would be valuable: a major concern for participants was that professionals might share information unawares to them. For this reason, many participants wanted an agree/disagree choice about sharing. We are not recommending that HCPs give patients an explicit choice, but argue that upfront discussion would enable and empower patients to participate actively in conversations about their information. In such discussions, HCPs might highlight ways that sharing over non-disclosure can be beneficial for individual patients: our study and others suggest that family secrets might damage relationships, whereas informed relatives could support each other.^26^

### Limitations and further research

A possible limitation of our work is that we did not include an exhaustive review of ethical arguments surrounding consent, confidentiality and information-sharing. We also based our conclusions on the analysis of the views of a small set of participants, which we, as researchers, distilled and mediated. Nevertheless, as many empirical ethics researchers have argued,^27^ there are important insights to be gained from even small empirical research studies, and participants’ views can be weighed in the balance and contribute to the quality of ongoing ethical arguments.

More research could be useful, however, since our analyses and conclusions might have differed were other participants interviewed. Despite us asking clinicians and genetic counsellors to send information specifically to anyone who had withheld information or had their information shared without consent, no such person participated, meaning participants’ considerations about non-disclosures were mostly hypothetical. We also did not collect details such as socioeconomic status and educational level. Although we would not have been able to make conclusions about the impact of such factors on views using qualitative methodology, it might be worthwhile to explore these questions in future quantitative research. This is particularly since people with higher incomes have been shown as more likely than those with lower incomes to be concerned about confidentiality and to think that genetic information deserves special protections in medical records.^16^ Religion, age, gender, and marital status have too been shown to impact on views about sharing genetic test results.^28^ We additionally call for further research about the practicalities of sharing information.

## Conclusion

Our findings illuminate views about consent, confidentiality and information-sharing and show overall support for the relational joint account. We hope our conclusions help HCPs gain a better insight into how to approach consent and confidentiality—not just for patients who explicitly refuse to share, but for all patients—and encourage them to take appropriate familial sharing as the starting point. In our wider project, we will be exploring how HCPs’ views compare with our participants’ views that genetic information should be disclosed and highlighting what, if any, consensus exists among them.

## Supplementary Material

Web supplement 1

Web supplement 2

## Footnotes

[i] We use the term ‘participant’ to refer to the 33 participants in the current study and ‘patient’ to refer generally to patients in the NHS. In addition, when we talk about relatives, we intend to mean that they were not the HCPs’ patients.

## References

[1] Widdows H Between the individual and the community: the impact of genetics on ethical models. New Genet Soc 2009:28:173–88. 10.1080/14636770902901611

[2] Clarke A, Richards M, Kerzin-Storrar L, et al Genetic professionals’ reports of nondisclosure of genetic risk information within families. Eur J Hum Genet 2005;13:556–62. 10.1038/sj.ejhg.5201394 15770225

[3] Parker M Scaling ethics up and down: moral craft in clinical genetics and in global health research. J Med Ethics 2015;41:134–7. 10.1136/medethics-2014-102303 25516955 PMC4283669

[4] GMC. Confidentiality. London: General Medical Council, 2009;67–9.

[5] BMA. Confidentiality as part of a bigger picture. London: BMA, 2005.

[6] ASHG statement. Professional disclosure of familial genetic information. The American Society of Human Genetics Social Issues Subcommittee on Familial Disclosure. Am J Hum Genet 1998;62:474–83. 10.1086/301707 9537923 PMC1376910

[7] Gilbar R Communicating genetic information in the family: the familial relationship as the forgotten factor. J Med Ethics 2007;33:390–3. 10.1136/jme.2006.017467 17601865 PMC2598139

[8] Parker M, Lucassen AM Genetic information: a joint account? BMJ 2004;329:165–7. 10.1136/bmj.329.7458.165 15258076 PMC478234

[9] British Society for Genetic Medicine/Joint Committee on Medical Genetics. Consent and confidentiality in genetic practice. London, Royal College of Physicians and Royal College of Pathologists, 2011.

[10] Doukas DJ, Berg JW The family covenant and genetic testing. Am J Bioeth 2001;1:3–10. 10.1162/152651601750417784 11954587

[11] Macklin (1992) Privacy control of genetic information. In: Annas GJ, Elias R, eds. Gene mapping: using law and ethics as guides. New York: Oxford University Press:157–72.

[12] Foster C, Herring J, Boyd M Testing the limits of the ‘joint account’ model of genetic information: a legal thought experiment. J Med Ethics 2015;41:379–82. 10.1136/medethics-2014-102142 24965717

[13] Cohen LL, Stolerman M, Walsh C, et al Challenges of genetic testing in adolescents with cardiac arrhythmia syndromes. J Med Ethics 2012;38:163–7. 10.1136/medethics-2011-100087 21955955 PMC3258368

[14] Lacroix M, Nycum G, Godard B, et al Should physicians warn patients’ relatives of genetic risks? CMAJ 2008;178:593–5. 10.1503/cmaj.070956 18299550 PMC2244654

[15] Benkendorf JL, Reutenauer JE, Hughes CA, et al Patients’ attitudes about autonomy and confidentiality in genetic testing for breast-ovarian cancer susceptibility. Am J Med Genet 1997;73:296–303. 10.1002/(SICI)1096-8628(19971219)73:3<296::AID-AJMG13>3.0.CO;2-E 9415688

[16] Plantinga L, Natowicz MR, Kass NE, et al Disclosure, confidentiality, and families: experiences and attitudes of those with genetic versus nongenetic medical conditions. Am J Med Genet 2003;119C:51–9. 10.1002/ajmg.c.10006 12704638 PMC4809525

[17] Akpinar A, Ersoy N Attitudes of physicians and patients towards disclosure of genetic information to spouse and first-degree relatives: a case study from Turkey. BMC Med Ethics 2014;15:39 10.1186/1472-6939-15-39 24885495 PMC4029893

[18] Kohut K, Manno M, Gallinger S, et al Should healthcare providers have a duty to warn family members of individuals with an HNPCC-causing mutation? A survey of patients from the Ontario Familial Colon Cancer Registry. J Med Genet 2007;44:404–7. 10.1136/jmg.2006.047357 17551085 PMC2740891

[19] Pentz RD, Peterson SK, Watts B, et al Hereditary nonpolyposis colorectal cancer family members’ perceptions about the duty to inform and health professionals’ role in disseminating genetic information. Genet Test 2005;9:261–8. 10.1089/gte.2005.9.261 16225406 PMC4007282

[20] Shkedi-Rafid S, Dheensa S, Crawford G, et al Defining and managing incidental findings in genetic and genomic practice. J Med Genet 2014;51:715–23. 10.1136/jmedgenet-2014-102435 25228303

[21] Mays N, Pope C Rigour and qualitative research. Bmj 1995;311:109–12. 10.1136/bmj.311.6997.109 7613363 PMC2550154

[22] Braun V, Clarke V What can “thematic analysis” offer health and wellbeing researchers? Int J Qual Stud Health Well-being 2014;9:26152 10.3402/qhw.v9.26152 25326092 PMC4201665

[23] Brock DW Genetics and confidentiality. Am J Bioeths 2001;1:34–5. 10.1162/152651601750417946 11954591

[24] Australian Law Reform Commission. Confidentiality and Admissibility. Retrieved 9/3/2015 http://www.alrc.gov.au/publications/22.%20Confidentiality%20and%20Admissibility/fdr-and-family-counselling-confidentiality

[25] Stol YH, Menko FH, Westerman MJ, et al Informing family members about a hereditary predisposition to cancer: attitudes and practices among clinical geneticists. J Med Ethics 2010;36:391–5. 10.1136/jme.2009.033324 20605992

[26] Klitzman R Am I my genes? Oxford: Oxford University Press, 2012.

[27] Kerruish N, McMillan JR Parental reasoning about growth attenuation therapy: report of a single-case study. J Med Ethics 2015;41:745–9. 10.1136/medethics-2013-101913 25858291

[28] Gilbar R, Shalev S, Spiegel R, et al Patients' attitudes towards disclosure of genetic test results to family members: the impact of patients' sociodemographic background and counseling experience. J Genet Counsel 2015; 10.1007/s10897-015-9873-1 26371363

