# Supplementary material for: ‘Is this knowledge mine and nobody else's? I don't feel that.’ Patient views about consent, confidentiality and information-sharing in genetic medicine
Source: J Med Ethics. 2016 Jan 7;42(3):174–9. doi: 10.1136/medethics-2015-102781 (PMC4789809; doi:10.1136/medethics-2015-102781)
Supplement: Web supplement 1 [file medethics-2015-102781-s1.pdf]

Supplementary Figure 1: how we framed the project's purpose

Participant information sheet

Purpose of the study

A genetic test in one person can sometimes suggest that other family members also have an increased chance of developing a condition. When this happens, there may be questions about if, when and how these family members should be told about the information.

In our research, we want to understand what people think about whether or not to share their genetic information with family members and other healthcare professionals. In particular, we want to find out what you think about the consent process and about confidentiality in genetic medicine. We hope that the findings from our research will help healthcare professionals and family members communicate better in the future.

Interview pre-amble

"We're interested in people's views about family. More specifically, we're looking at how people feel about privacy and confidentiality regarding family illness, how families and the NHS manage information, and whether doctors and nurses should have more of a role in sharing information with patients' families about their risks."
