# Supplementary material for: ‘Is this knowledge mine and nobody else's? I don't feel that.’ Patient views about consent, confidentiality and information-sharing in genetic medicine
Source: J Med Ethics. 2016 Jan 7;42(3):174–9. doi: 10.1136/medethics-2015-102781 (PMC4789809; doi:10.1136/medethics-2015-102781)
Supplement: Web supplement 2 [file medethics-2015-102781-s2.pdf]

Table 2: Additional and supportive quotes

1i: Genetic information is familial	P5: Whoever they are—distant cousins or whatever—they've every right to know because it concerns them and it concerns their family. [possible Lynch,F]<br><br>P17: For me, it wouldn't bother me whether it was somebody I hated the sight of, I wouldn't like to see anybody go through what I went through, and it's as simple as that. If that DNA test is going to help somebody, let them use it. [Lynch,M]<br><br>P1: In all honesty it's just hindering them. It's not going to help me. It's not going to solve the disease for me. It's not going to create any treatment benefits for me to not let them know. I guess that's the be-all and end-all, the way I look at is if it's going to benefit them, do I have a moral obligation to at least let them know […] 'look, this is something to look out for'. Whether we're close or not, we're still related; I can't sever the blood tie, and so that's never going to happen. We're always going to have that relationship. We may not have the fellowship, but we'll always have that relationship. [FAP,M]<br><br>P28: Is this knowledge mine and nobody else's? I don't feel that. I feel knowledge is free, and I can understand about confidentiality, but I'm trying to think, really, what confidential, what sensitive information would make me feel I didn't want people to know? And I suppose, I don't know. [BRCA,F]<br><br>P2: I don't actually think of [my diagnosis as confidential], because yes my personal information's confidential. But we have the same genes for goodness sake; we've come from the same pool. [Alport,F]<br><br>P6: It is science isn't it? It's general research. I mean, whatever we've got, it's personal. Even if 5,000 others have got it, but we don't own that. I don't see us as owning that knowledge privately, at all. I see it as general research, the results of general research […] I don't see it as personal knowledge; I see it as general science, information to help the world go forward and to keep the world healthy. [Alport,F]<br><br>P24: I would feel so betrayed not knowing. [BRCA,F]<br><br>P18: I think it would take a particularly selfish person not to want to pass that on to their family members, but I can admit it could easily happen, because as I say my sister could have contacted me directly and chose not to, although she did luckily pass it on to the medical professional. [BRCA,F]

Table 2: Additional and supportive quotes

1ii: A tricky situation, but confidentiality is “by-the-by”	P3: I think they still should somehow manage to inform the other relatives that they’re at risk, because it’s very selfish of someone to say ‘no, I don’t want anybody else to know’. Sometimes you have fallen out, but there’s nothing to say that maybe in time they may come to terms and come back together. Families, it evolves all the time. [Alport,F]<br><br>P12: I think it’s almost a duty, whether you’re close to your family or not. If there was any possible way of you helping them, in whatever way, then it should be done. I think it’s incredibly selfish not to and you don’t want anybody to go through that. [possible BRCA,F]<br><br>P25: I think it’s completely selfish not to give somebody choice, and if it was me and my sister hadn’t told me and I had found out, I’d be very upset. And if that’s the case, then when the GP, I think the GP should make that person aware really of the possibility. [BRCA,F]<br>P13: What is greater, [a person’s] right to privacy or the[ir] daughters’ right to their lives and their health? I think that then becomes more important than that person’s right to privacy. [Cardiomyopathy,M]<br><br>P7: I don’t think ultimately my confidentiality or my privacy should be respected over the risk of somebody having long-term health issues. [Hemochromatosis,M]<br><br>P14: If it means allowing somebody to be treated earlier and maybe surviving, then that person’s right to life is more than that person’s right to privacy. [HCPs should] be interested in trying to help people, trying to make sure that they were you know alive enough to have those opinions, rather than the opposite. [BRCA,M]<br><br>P30: I don’t think they should be able to say your cousin Derek has whatever syndrome, because that is directly going against that person’s wishes, and they’ve got a right to their privacy [Lynch,F]<br><br>P10: I don’t know whether I think it would be a great idea maybe to say necessarily who, because some people might feel more private about it than I do. [BRCA,F]<br><br>P19: They probably wouldn’t want to let that person know that it’s your brother or sister or your cousin. [Lynch,M]

Table 2: Additional and supportive quotes

	P3: I think that [HCPs telling other family members] would be fine, because it's, if it's just your family and it's gone out your family tree, and you didn't know they'd got it and they didn't know you'd got it, you could be sharing information with them. If they didn't know and you didn't know, you're both going along on your own paths and perhaps you know you've got stories or tips that you could use together. If you didn't see them that much or they were quite a distant relative you could lose all that and they could then be more ill or vice versa and they perhaps could have been helped if you'd known about each other [Alport,F]
1iii: Broad harms justify disclosure	P26: If it's a much more debilitating condition, where somebody may not live say into their thirties, and you're basically continuing to spread this gene around in effect, and you're bearing children who may have this disease, you may want to think twice or go through a different avenue. [BRCA,F]<br><br>P24: It would completely, it really alters your life. You definitely have to make different life choices, like I need to have children young and you have to prepare for these things, and if you don't know it, it completely could ruin your life. I mean obviously you don't want to hurt the confidentiality of [the patient], but at the same time you have to keep in mind that there is this other person and their life is also greatly affected by this. [BRCA,F]<br><br>P10: It could cause trauma. I mean people get post-traumatic stress disorder from all sorts of experiences and it affects the physiological and psychological health, so it's imperative that people have all the information that they can about things that might affect their health. So the professional should be entitled to provide that information. [BRCA,F]
2i: Choice has upsides and downsides	I: Do you feel like you want the option yes or no?<br>P17: No. No, as long as it's going to help somebody. Because you might get some part of the families who don't get on, and say 'I ain't giving him my test results', and that's, I don't know, it's a hard thing. [Lynch,M]<br><br>P19: A [agree/disagree] choice is better, but the first point that you mentioned about my cousin not letting them know [referring to the non-disclosure scenario], from that point of view, I would say default [sharing] in some cases is better, because it's just blanket coverage, so if I know something's wrong with me, all my family comes to know [and] there is no possibility of me keeping a secret from my family. [Lynch,M]

Table 2: Additional and supportive quotes

	P13: For me, I guess you just need that mechanism there in case you have problems in your family and you may have fallen out with someone or whatever, and for me, even that wouldn't have changed it, but I can see why people would want that. And I guess it just is nice to know what's going to happen with your information. I still would like to have the option I guess. I don't know why that is, because I would always say yes. [Cardiomyopathy,M]<br><br>P10: I don't think anybody is entitled to know anything about me unless I choose to allow them […] If I were secretly autistic or schizophrenic or had bipolar [disorder] I wouldn't want to share that. [BRCA, F]<br><br>P29: Anything to help the test being done quicker or information being shared quicker, especially when it's something quite serious, so no, I wouldn't want to hinder anybody. [BRCA,F]<br><br>P6: It's really important that these things are out in the open. And if somebody is going to be sick, then goodness me I've seen in it in my family, the earlier you get it the better. The reason I lost siblings was because they didn't know what it was. [Alport,F].
2ii: Confusion about the law	P6: I still am oblivious to how [my genetic information is] being used. But that raises a concern for me that someone somewhere will find a lawyer who will say hang on, you took blood from me, you did a genetic test without my authorisation, you've invaded my human rights, and then, it sounds ridiculous, but my concern is that the NHS are then forced to pay out to someone, unnecessarily. So I think there should have been more information given; an explanation- 'we take the blood, we do this test, your blood is disposed of, that test then sits on a database that identifies this…' [Haemochromatosis,M]
